# *Larrea ameghinoi* Speg. (Zygophyllaceae) “Jarilla Rastrera”: UHPLC-ESI-QTOF-MS Analysis, Antioxidant, Antimicrobial Properties, and Inhibition of Enzymes of Interest to Human Health

**DOI:** 10.3390/antiox15060668

**Published:** 2026-05-26

**Authors:** Jessica Gómez, Silvana M. Sede, Belén Ariza Sampietro, Daniel Zaragoza-Puchol, María Elisa Bressan Merlo, Duilio Caballero, Beatriz Lima, Alejandro Tapia, Mario J. Simirgiotis

**Affiliations:** 1 Instituto de Biotecnología-Instituto de Ciencias Básicas-Departamento de Ingeniería Agronómica, Universidad Nacional de San Juan (UNSJ), San Juan J5400ARL, Argentina; jegomez@unsj.edu.ar (J.G.); barizasam88@gmail.com (B.A.S.); josedanielzaragoza@gmail.com (D.Z.-P.); elisabressan2@gmail.com (M.E.B.M.); blima@unsj.edu.ar (B.L.); 2 Consejo Nacional de Investigaciones Científicas y Técnicas (CONICET), Ciudad Autónoma de Buenos Aires C1425FQB, Argentina; ssede@darwin.edu.ar; 3 Instituto de Botánica Darwinion CONICET-ANCEFN Labardén 200, San Isidro B1642HYD, Buenos Aires, Argentina; 4 Laboratorio Hospital Marcial Quiroga, Av. Libertador General San Martín 5401 (O), Rivadavia CP 5407, San Juan, Argentina; duiliocaballero@gmail.com; 5 Instituto de Farmacia, Facultad de Ciencias, Campus Isla Teja, Universidad Austral de Chile, Valdivia 5090000, Chile; 6 Center for Interdisciplinary Studies on the Nervous System (CISNe), Universidad Austral de Chile, Valdivia 5090000, Chile

**Keywords:** phenolic characterization, antioxidant, antimicrobial, enzymes, lignans

## Abstract

*Larrea ameghinoi* Speg., an endemic species of Argentine Patagonia traditionally used in folk medicine to treat fever, stomach disorders, respiratory conditions, back pain, and as an emmenagogue, among others, still remains chemically and biologically underexplored compared to the other four members of the genus. This study aimed to perform a comprehensive metabolomic characterization of methanolic extracts from two populations (EMLaSAO and EMLaMAQ) using ultra-high-resolution liquid chromatography coupled with electrospray ionization quadrupole time-of-flight mass spectrometry (UHPLC–ESI–QTOF–MS) and to evaluate their antioxidant, antimicrobial, and enzyme-inhibitory activities of relevance to human health. Thirty-three compounds were tentatively identified by extensive UHPLC–MS analysis, including flavones, two major lignans, and oleanane-type triterpenes. Both extracts exhibited high phenolic content (215–239 mg of gallic acid equivalents (GAE)/g extract) and strong free radical scavenging activity, as evidenced by 2,2-diphenyl-1-picrylhydrazyl (DPPH, EC_50_ ≈ 10 μg/mL), ferric-reducing antioxidant power (FRAP), and Trolox equivalent antioxidant activity (TEAC) assays. In addition, significant inhibition of butyrylcholinesterase (IC_50_ ≈ 50 μg extract/mL) and α-glucosidase, together with selective antibacterial activity against methicillin-sensitive and resistant *Staphylococcus aureus* (MIC = 125 μg extract/mL), were recorded. These findings suggest that *L. ameghinoi* possesses a distinctive phytochemical composition conferring multitarget bioactivity, differing from other Larrea species dominated by lignans such as nordihydroguaiaretic acid (NDGA) and its derivatives. Overall, this work supports the potential of *L. ameghinoi* as a novel source of bioactive metabolites for managing oxidative stress-related disorders and opportunistic infections. This warrants future in vivo studies investigating biological activities associated with oxidative stress and their relevance to human health.

## 1. Introduction

**Figure 1:**
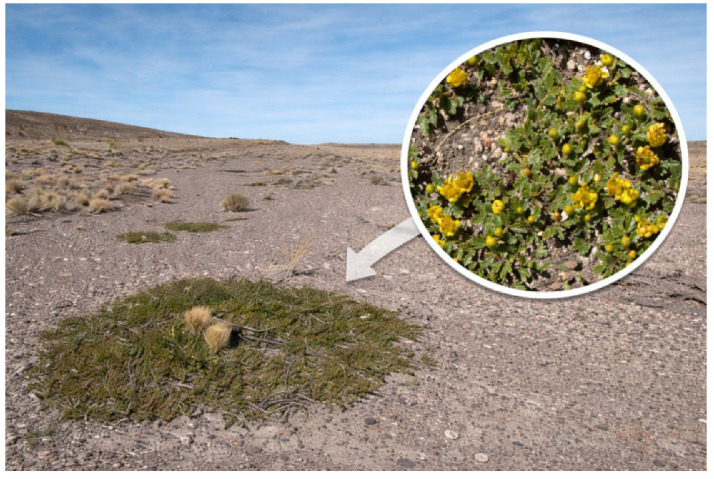
*L. ameghinoi* Speg. habit and typical habitat (Patagonian steppe).

Medicinal resinous species belonging to the genus Larrea Cav. (Zygophyllaceae) are distributed amphitropically in arid environments of Argentina, Chile, Bolivia, Peru, Mexico, and the southwestern United States. The genus is composed of five species: *Larrea ameghinoi* Speg., *L. cuneifolia* Cav., *L. divaricata* Cav., and *L. nitida* Cav., commonly known as “jarillas”, occurring in South America, while *L. tridentata* (DC.) Coville grows only in Mexico and the USA [1,2,3]. The species are used extensively in traditional medicine in Argentina for the treatment of injuries and bruises, and are a good disinfectant for wounds and a repellent against insects; they are also used for roof construction in rural areas and as a plant-based fuel for cooking [4]. Extracts and infusions of aerial parts of most species of the genus Larrea have displayed several biological activities, such as antimicrobial, antioxidant, enzyme-inhibitory, anti-inflammatory, and antitumor activities, among others [4]. A notable research outcome involving *Larrea divaricata* was the recent development of a natural lotion composed of jarilla and decaffeinated coffee extracts to stimulate hair growth, reduce hair loss, and permanently control dandruff. The said product has been registered and obtained a patent [5]. *Larrea ameghinoi* Speg., commonly known as “jarilla rastrera”(Figure 1), is a shrub endemic to Argentine Patagonia that lives in lowland areas from Neuquén to Chubut between 200 and 800 m above sea level [6]. It is a resinous, branchy, prostrate woody plant with twisted branches and measures approximately 10 cm in height. It flowers from October to the end of November, a period in which its yellow flowers can be seen. It is distinguished from the rest of the species of the genus by having subsessile leaves (5–8 × 2–3 mm), with 3–7 unequal, imparipinnate leaflets, with a smaller, fused terminal leaflet. Its fruit is a schizocarp, verrucose and brown-red in color, which has five mericarps that remain associated at maturity; the seeds are small, smooth, and kidney-shaped [7,8]. The plant adopts a cushion-shaped structure, which gives it the ability to thrive in areas exposed to the westerlies, the characteristic winds of Patagonia, even when this implies a reduction in exposure to sunlight. In addition to its cultural and symbolic importance, *L. ameghinoi* constitutes a promising source of bioactive compounds with potential benefits for human health. It is valued by indigenous peoples, and the uses of the species of this genus have been transmitted through popular knowledge. The bark and leaf are popularly used to treat different ailments in the form of various preparations, to reduce fever, stomach disorders, and respiratory conditions, to treat back pain, and as an emmenagogue since it stimulates and promotes menstrual flow. In addition, it soothes rheumatic pain due to its application in the form of a poultice [8].

To date, studies on the chemical characterization and potential biological activity of this species are limited.

The main goals and novelty of this work are the evaluation of the antioxidant and antibacterial effects, as well as the inhibition of enzymes of interest to human health, complemented by the complete polyphenolic metabolome profile by UHPLC-ESI-QTOF-MS analysis of the methanolic extracts of *L. ameghinoi* from Argentina.

## 2. Materials and Methods

### 2.1. Chemicals

Ultra-pure water, with total organic carbon (TOC) levels below 5 µg/L, was sourced using an Arium 126 61316-RO purification system coupled with an Arium 611 UV unit (Sartorius, Goettingen, Germany). High-purity methanol (HPLC grade) and mass spectrometry-grade formic acid were supplied by J. T. Baker (Phillipsburg, NJ, USA), while HPLC-grade chloroform was provided by Merck (Santiago, Chile). Commercial Folin–Ciocalteu (FC) reagent, 2,2-diphenyl-1-picrylhydrazyl (DPPH), ferric chloride hexahydrate, 2,4,6-tris(2-pyridyl)-s-triazine, trolox, quercetin, gallic acid, and dimethyl sulfoxide (DMSO) were purchased from Sigma-Aldrich Chem. Co. (St Louis, MO, USA). Nordihydroguaiaretic acid (NDGA), 3′-methyl-nordihydroguaiaretic acid (MNDGA), and other lignans previously isolated from Larrea nitida and some HPLC standards were acquired from Sigma-Aldrich Chem. Co. (St Louis, MO, USA) or Extrasynthèse (Genay, France) were used for UHPLCMS analysis.

### 2.2. Plant Material, Methanolic Extracts, and the Total Phenol and Flavonoid Content

Considering the vulnerability of *L ameghinoi*, aerial parts (10 g) were collected in two locations in the province of Rio Negro, Maquinchao (MAQ) and a location near Las Grutas (SAO). The plant samples were dried at room temperature (25 °C) and stored in conditions devoid of light and heat. A voucher specimen for each locality has been deposited in the herbarium of Instituto de Botánica Darwinion (CONICET-ANCEFN) under the reference numbers Sede & Calcagno 885 (SAO) and Sede & Abraham 915 (MAQ).

From dried and ground plant material, the methanolic extracts of each sample were prepared as follows: 10 g of plants from SAO and MAQ were weighed, and successive extractions were carried out with methanol under sonication at a temperature of 30 °C. The process was repeated twice, each time. The resulting extracts were subjected to a filtration stage to eliminate solid residues. Subsequently, they were concentrated at reduced pressure in a rotary evaporator until constant weight was obtained, thus obtaining both methanolic extracts called EMLaSAO and EMLaMAQ, which yielded 12.79% and 16.69% *w*/*w*, respectively. The extracts were stored at −20 °C until they were analyzed via UHPLC–PDA–QTOF-MS, as well as for the spectrophotometric quantification of phenolics and flavonoids, and in vitro assays.

The total phenol content [9] and the flavonoid content were expressed as milligrams of gallic acid equivalents (GAE) or milligrams of quercetin equivalents (QE) per gram of EMLaSAO and EMLaMAQ (mg GAE/g EMLaSAO and EMLaMAQ), respectively. The values were obtained in quadruplicate using a Multiskan FC Microplate Photometer (Thermo Scientific, Waltham, MA, USA). The results are expressed as mean ± SD.

### 2.3. In Vitro Studies

#### 2.3.1. Antioxidant Activity

##### Radical Scavenging Capacity Assay of 2,2-Diphenyl-1-Picrylhydrazyl (DPPH)

The free radical scavenging activity of EMLaSAO and EMLaMAQ was assessed using the DPPH assay. The concentration required to achieve 50% radical scavenging (EC50) was determined from a graph plotting inhibition percentage at 517 nm against concentrations of EMLaSAO and EMLaMAQ. Catechin (Sigma-Aldrich, ≥98%, St. Louis, MO, USA) served as the reference compound (EC_50_ 4.1 μg/mL). Each test was conducted in quadruplicate [10].

##### Ferric-Reducing Antioxidant Power Assay (FRAP)

Results from the FRAP assay were calculated using linear regression based on the FRAP–Trolox calibration curve and are expressed as milligrams of Trolox equivalents per gram of EMLaSAO and EMLaMAQ (mg ETrolox/gof EMLaSAO and EMLaMAQ). All tests were conducted in quadruplicate [11].

##### Trolox Equivalent Antioxidant Activity Assay (TEAC)

The TEAC assay results were obtained through linear regression from a calibration curve created using Trolox concentrations ranging from 0 to 1 mM and are reported as milligrams of Trolox equivalents per gram of EMLaSAO and EMLaMAQ (mg ETrolox/g of EMLaSAO and EMLaMAQ. Each test was performed in quadruplicate [12].

### 2.4. Enzymatic Activity

#### 2.4.1. Acetylcholinesterase and Butyrylcholinesterase Inhibitory Bioassay

Cholinesterase inhibitory activities were evaluated according to Ellman et al. (1961) [13] with some minor modifications. Enzyme inhibitory activity was calculated as a percentage compared to an assay using a buffer without any inhibitor. The values were expressed as half-maximal inhibitory concentration IC_50_ (µg/mL for extracts and galantamine), and were calculated as means ± SD of 3 individual determinations. The galantamine concentrations used to calculate the IC_50_ values were 0–1, 0.5, 1, 1.5, and 2 µg/mL in both AChE and BuChE assays, while for the EMLaSAO and EMLaMAQ extracts, they were 25, 50, 100, 150, 200, 250, and 300 µg/mL, respectively. The enzymes AChE and BuChE from Sigma-Aldrich were used. Galanthamine (Sigma-Aldrich) was used as a positive control in both assays.

#### 2.4.2. Amylase and Glucosidsase Inhibition

The amylase inhibition was implemented by agreeing to the method previously described by [14]. On the other hand, the glucosidase inhibition assay was evaluated according to the method described by [15]. Both assays are shown as IC_50_ in µg/mlas means ± SD.

### 2.5. Antibacterial Activity

#### 2.5.1. Antibacterial Susceptibility Testing

The minimum inhibitory concentration (MIC) and minimum bactericidal concentration (MBC) of EMLaSAO and EMLaMAQ and the antibiotic Imipecil (Imipenem, from Laboratory Northia, Buenos Aires, Argentina) were determined by broth microdilution techniques, according to Clinical and Laboratory Standards Institute (CLSI) [16]. Both extracts were tested from 1000 to 0.98 µg/mL using an inoculum of each bacterium adjusted to 5 × 10^5^ cells with colony-forming units (CFU)/mL. The following strains from the American Type Culture Collection (ATCC, Rockville, MD, USA) and clinical isolates from Laboratorio de Micro-biología, Hospital Marcial Quiroga, San Juan, Argentina (MQ) were employed: *Staphylococcus aureus* methicillin-sensitive ATCC 25923 (MSSA), *Staphylococcus aureus* methicillin-resistant ATCC 43300 (MRSA), *Staphylococcus aureus* methicillin-resistant-MQ1, *Staphylococcus aureus* methicillin-resistant-MQ2, *Salmonella* sp-MQ3, and *Escherichia coli* ATCC 25922.

#### 2.5.2. Antifungal Susceptibility Testing

Minimum inhibitory concentration (MIC) of EMLaSAO and EMLaMAQ was determined using broth microdilution techniques, according to the guidelines of the CLSI [17]. For the development of the essay, stock solutions of EMLaSAO and EMLaMAQ were two-fold diluted with RPMI medium from 1000 to 0.98 µg/mL (final volume 100 µL and final dimethyl sulfoxide (DMSO) concentration ≤ 1%). A 100 µL volume of inoculum suspension was added to each well, except for the sterility control well, where sterile water was added instead. Ketoconazole (Sigma-Aldrich) was used as a positive control. Inoculum of cell or spore suspensions was obtained according to reported procedures and adjusted to 1–5 × 10^3^ cells/spores with colony-forming units (CFU)/mL. The methodology for the determination of MIC_100_ and MFC has been reported in detail in [18].

The following strains from the ATCC, MQ, and from CEREMIC (CCC), Reference Center in Mycology, Faculty of Biochemical and Pharmaceutical Sciences, Suipacha 531, 2000-Rosario, Argentina, and from were employed: *Candida albicans*-MQ 1924, *Candida glabrata* MQ1234, *Candida parapsilosis* MQ3, *Candida tropicalis* CCC 131-2000, and *Cryptococcus neoformans* ATCC 32264.

### 2.6. Ultra-High-Resolution Liquid Chromatography Analysis of EMLaSAO and EMLaMAQ

The analysis of EMLaSAO and EMLaMAQ was performed using a UHPLC-ESI-QTOF-MS system, integrated by a UHPLC Ultimate 3000 RS with Chromeleon 6.8 software (Dionex GmbH, Idstein, Germany), and a Bruker maXis ESI-QTOF-MS. The analysis of both extracts was performed on an Acclaim Thermo 5 µm C18 80 Å (150 mm × 4.6 mm) column at a flow rate of 1.0 mL/min, using a two-solution system: eluent (A) 0.1% formic acid in water and eluent (B) 0.1% formic acid in acetonitrile. The solution elution program was as follows: 1% isocratic B (0–2 min), 1–5% B (2–3 min), 5% isocratic B (3–5 min), 5–10% B (5–8 min), 10–30% B (8–30 min), 30–95% B (31–38 min), and 1% isocratic B (39–50 min). ESI-QTOF-MS experiments in negative ion mode were recorded, and the scanning range was between 100 and 1200 *m*/*z*. For the analysis, 5 mg of each extract was dissolved in 2 mL of methanol, passed through a polytetrafluoroethylene (PTFE) filter, and 10 µL was injected into the apparatus. MS data were analyzed using Bruker Data Analysis 4.0 (Bruker Daltonik GmbH, Bremen, Germany) and ACD lab spectrum processor (New York, NY, USA) software v2013.

### 2.7. Statistical Analysis

The results were analyzed with GraphPad Prism (GraphPad Software Inc., v.9, San Diego, CA, USA). The comparisons between two groups were conducted using Student’s t-test, whereas the comparisons between three groups were assessed using one-way ANOVA followed by Duncan or Newman–Keuls post hoc tests. The in vitro results were expressed as mean standard deviation (SD). All differences were considered significant when *p* < 0.05.

## 3. Results

**Figure 2:**
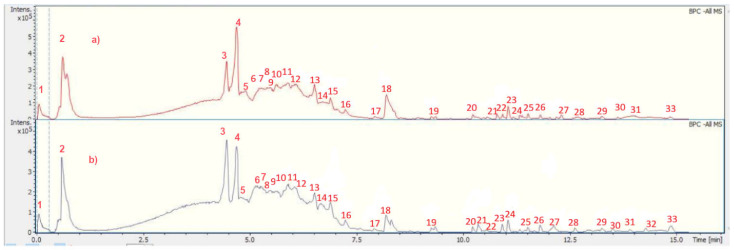
UHPLC Q-TOF-TIC chromatogram of EMLaMAQ (**a**) and EMLaSAO (**b**).

**Figure 3:**
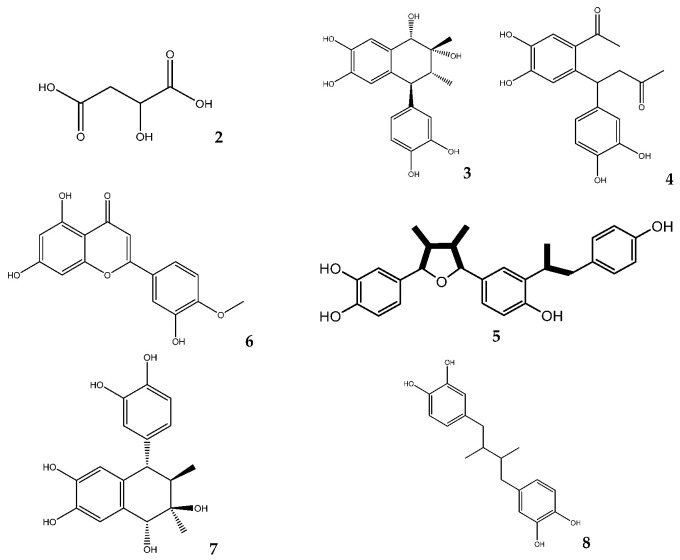

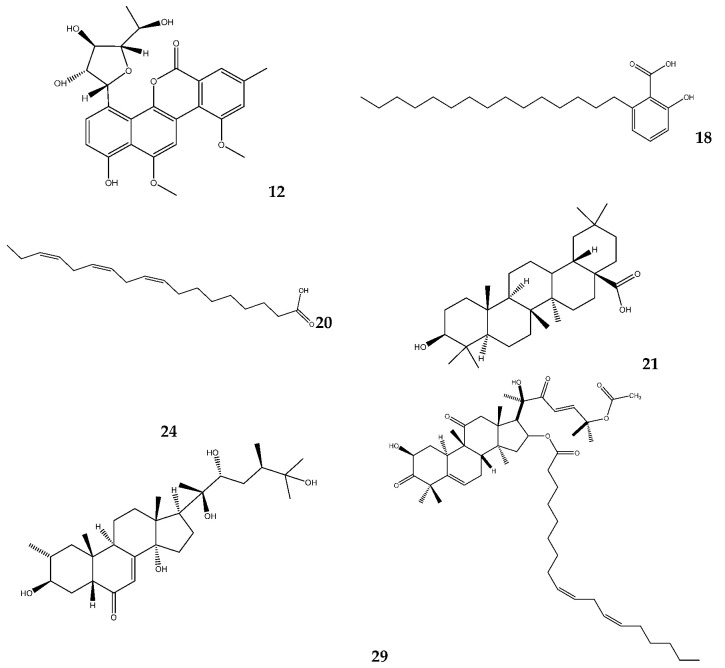
Molecular structures of representative compounds identified in EMLaMAQ and EMLaSAO.

The characterization strategy using UHPLC-ESI-QTOF-MS of chemical compounds present in EMLaSAO and EMLaMAQ involved, initially, data analysis carried out using Metaboscape 4 software (Bruker, Billerica, MA, USA), a tool that allows the identification of metabolites based on their mass, fragmentation pattern, and isotopic pattern. The exhaustive UHPLC-ESI-QTOF-MS analysis in negative mode revealed, in this first analysis, the presence of 33 peaks (2–33) in EMLaSAO and EMLaMAQ, corresponding to 24 tentatively identified compounds (**2**,**3**,**4**,**5**,**6**,**7**,**8**,**9**,**10**,**11**,**12**,**15**,**16**,**18**,**19**,**20**,**21**,**22**,**23**,**24**,**25**,**28**,**29**); 8 unknown compounds (**14**,**15**,**26**,**27**,**30**–**33**) and the peak 1 (internal standard). These included flavones, aromatics, terpenes, sterols, furans, and several fatty acids, all of which are tentatively identified here for the first time in *L. ameghinoi*. The identification strategy employed involved spiking experiments with available standards. Additionally, a comprehensive search of several databases, including MassBank of North America (MONA) and Metaboscape, was performed, along with the use of specific software such as Data Analysis 4.0 (Bruker Daltonik GmbH, Bremen, Germany) and ACD/Spectrum Processor (ACD/Labs, Toronto, Canada), as well as previous reports on the genus Larrea [18,19]. The high-resolution UHPLC–PDA–QTOF analysis results for metabolite identification in EMLaSAO and EMLaMAQ are displayed in Figure 2 and Table 1. Furthermore, the chemical structures of some of the main compounds identified are shown in Figure 3.

Table 2 shows the antioxidant properties and total phenolic and flavonoid content of EMLaSAO and EMLaMAQ, which were identified using standardized colorimetric methods and following standardized protocols previously reported in research on the medicinal flora of Argentina and Chile.

The results obtained from the enzymatic inhibitory activity evaluation of EMLaSAO and EMLaMAQ indicate that EMLaSAO exhibited greater inhibitory activity against AChE compared to EMLaMAQ, while both extracts showed similar inhibition of BChE (Table 3). Regarding digestive enzymes, EMLaSAO and EMLaMAQ demonstrated higher inhibitory efficacy against glucosidase.

The plant extracts EMLaSAO and EMLaMAQ from *L. ameghinoi* exhibited selective antimicrobial activity (Table 4), with EMLaSAO showing the best performance, particularly against Gram-positive *Staphylococcus aureus* strains, with MIC values ranging from 125 to 250 µg/mL. In contrast, activity against Gram-negative bacteria was weak or absent.

## 4. Discussion

In this article, the enzymatic inhibition, antioxidant and antibacterial effects, and polyphenolic profile of the methanolic extract of *L. ameghinoi* from Argentina are reported for the first time, supporting the potential of the species as a sustainable source of pharmacologically relevant biomolecules. While other species of the genus, including *L. divaricata, L. cuneifolia,* and *L. nitida,* have been extensively studied and associated with diverse biological activities, studies on *L. ameghinoi* remain limited. In this work, methanolic extracts from two Argentine populations of *L. ameghinoi* (SAO and MAQ) showed comparable total phenolic content (239.5 and 215.6 mg GAE/g of extract, respectively), with significantly higher flavonoid content in SAO (28.44 mg QE/g vs. 15.28 mg QE/g). Both extracts exhibited strong antioxidant activity, with IC_50_ values of 10 µg/mL in the DPPH assay, comparable to those of reference compounds such as catechin and quercetin. Consistent results were observed in the FRAP and TEAC assays, with no significant differences between the two samples. In a recent report, resins from *Larrea divaricata* and *L. nitida* showed strong DPPH radical scavenging with values around 8.4 µg resin/mL [4], similar to that exhibited by *L. ameghinoi* extracts (10 µg/mL). The Larrea species that grow in Argentina, including *L. cuneifolia*, *L. divaricata*, and *L. nitida*, are characterized by a high lignan content, notably NDGA and 3′-MNDGA, as well as their respective isomers, which have been identified as markers of these species and are partly responsible for the strong antioxidant and antimicrobial activity of their polar exudates and extracts. Additionally, the synergistic antifungal activity of some of these species, such as *Larrea nitida*, has also been associated with the content of the marker lignans NDGA and 3′-MNDGA [20]. Regarding the estimated chemical composition of EMLaMAQ and EMLaSAO, UHPLC Q-TOF-TIC chromatograms support that both populations stand out for the presence of three major compounds: malic acid (2), 1,2,4-trihydro-1-(3,4-dihydroxyphenyl)-2,3-dimethyl-3,4,6,7-naphthalenetetrol (3), and 4-(2-acetyl-4,5-dihydroxyphenyl)-4-(3,4-dihydroxyphenyl)butan-2-one (4). Unlike other species of the genus Larrea, both samples show relatively low content of key marker compounds of the genus, such as NDGA (8) and one of its isomers (11), while 3′-MNDGA and its isomers were not detected in the present analyses.Recently, it has been reported that the ability of phenolic compounds as free radical scavengers depends on the quantity and position of the hydroxyl and methoxy groups in their molecules. Additionally, compounds with a catechol group rather than a single hydroxyl group at position 4 have higher reducing capacities, as is the case for caffeic acid compared with p-coumaric acid, and for 3,4-dihydroxybenzoic acid compared with 4-hydroxybenzoic acid. One suggested justification is that stabilizing the phenoxyl radical through an intramolecular hydrogen bond enhances antioxidant activity [21]. Considering the solid and recent bibliography mentioned above, the potent DPPH radical scavenging activity of both samples could be partly explained by the predominant presence of compounds **3** and **4**, characterized by two catechol groups in their structures. A contribution from other compounds present in smaller proportions, which also have catechol systems in their structures, such as compounds **5**, **6**, and **7**–**11**, is also expected. Compounds **3**, **4**, and **7** have recently been reported from the aerial parts of *Larrea tridentata*, along with an evaluation of their cytotoxic activity. Compound **3**, identified as (1R,2R,3R,4S)-1,2,4-trihydro-1-(3,4-dihydroxyphenyl)-2,3-dimethyl-3,4,6,7-naphthalenetetrol, has been reported to exhibit cytotoxic activity in HL-60 cells with IC_50_ values of 16 ± 0.95 µM [19]. The flora of Argentina, including various genera of Asteraceae, has attracted attention due to its potential benefits in preventing chronic or non-communicable diseases, such as chronic respiratory diseases, cardiovascular diseases, diabetes, and cancer. These conditions, along with major degenerative pathologies (e.g., Parkinson’s and Alzheimer’s diseases), are leading causes of mortality worldwide, making the search for natural preventive alternatives a priority [22,23]. In this study, *L. ameghinoi* extracts showed low AChE inhibition but moderate BChE inhibition (IC_50_ ≈ 49–50 µg/mL), suggesting potential selective therapeutic applications. Previous studies have demonstrated cholinesterase inhibitory activity in other Larrea species, such as *L. tridentata*, with this activity being associated with species-specific marker compounds like NDGA (compound **8**) [24]. Furthermore, antioxidant compounds from dietary sources can neutralize reactive oxygen species, which are key mediators of functional decline and neuronal damage in Alzheimer’s disease, Parkinson’s disease, and other neurodegenerative disorders [25]. This suggests a mechanistic link between the antioxidant properties of the extracts and their ability to inhibit cholinesterase enzymes, supporting a potential neuroprotective effect. Overall, the biological effects observed for *L. ameghinoi* methanolic extracts can plausibly be explained by the synergy between phenolic metabolites (particularly flavones) and terpenoid/triterpenoid compounds detected by UHPLC. Among polyphenols, flavones stand out due to their potent electron-donating capacity, as well as their ability to modulate pro-oxidant targets, specifically diosmetin (compound **6**) and related analogs, which exhibit potent antioxidant activity and a multitarget profile relevant for neuroprotection (AChE/BuChE inhibition and Cu^2+^ chelation), consistent with the cholinesterase inhibition recorded in our enzymatic assays [26,27]. Similarly, long-chain phenols such as anacardic acids (compounds **18** and **19**) possess both antioxidant and anticholinesterase properties [28], providing an additional mechanistic link between phenolic content and the AChE/BuChE inhibitory effects observed in this study. It has recently been reported that anacardic acid exhibits anti-proliferative, pro-apoptotic, anti-inflammatory, and antinociceptive actions and also reduces oxidative stress in acute experimental models, suggesting compound **19** as a promising natural compound of interest for human health [29,30]. The results shown by *L ameghinoi* suggest potential and justify further research aimed at identifying the active principles responsible for the demonstrated biological activities. We are currently isolating and purifying the major compounds reported here, which will allow us to quickly evaluate their real potential in the biological activities reported here, and further investigate enzyme inhibition studies, including butyrylcholinesterase, and conduct molecular modeling studies to estimate the real potential against this enzyme and others.

Regarding other biological activities, the antimicrobial activity of EMLaSAO and EMLaMAQ was evaluated via MIC, MBC, and MFC (Table 4) and ranked according to the criteria reported in references [31,32]. EMLaSAO showed marked activity against Gram-positive bacteria, including methicillin-sensitive and methicillin-resistant Staphylococcus aureus (MIC = 125 µg/mL; MBC = 250 µg/mL), whereas Gram-negative bacteria were less susceptible (MIC = 1000 µg/mL), indicating a bacteriostatic effect. These results align with previous observations in other Larrea species. Zampini et al. (2007) [33] reported high activity of ethanolic extracts of *L. divaricata* and *L. cuneifolia* against *Escherichia coli* (MIC = 50 µg/mL), while Moreno et al. (2020) [34] observed moderate effects for *L. nitida*, *L. divaricata*, and *L. cuneifolia* (MIC = 800 µg/mL; MBC > 1000 µg/mL). Similarly, Gómez et al. (2021) [4] demonstrated that resinous exudates from *L. divaricata* and *L. nitida* effectively inhibited *E. coli* MQ 586 (MIC = 62.5 µg/mL) and methicillin-sensitive and resistant *S. aureus* (MIC = 16–32 µg/mL). On the other hand, Martins (2013) [35] found that specific methanolic fractions of L. tridentata, particularly the ethyl acetate fraction, exhibited an MIC of 31.3 µg/mL against methicillin-resistant *S. aureus*, surpassing even tetracycline (64 µg/mL). These interspecies differences suggest that antibacterial potency is strongly influenced by the particular chemical profile of each species, as well as by the nature of active metabolites, such as lignans and phenolic compounds, which have been associated with antimicrobial activity in Larrea.

With respect to antifungal activity, both extracts displayed moderate inhibition against clinical Candida species, with EMLaSAO generally more active (MIC = 125–500 μg/mL) than EMLaMAQ (MIC = 250–1000 μg/mL). The most susceptible strain was *C. tropicalis* C131, for which EMLaSAO displayed an MFC of 500 μg/mL, while other yeasts did not show significant fungicidal effects. Compared with extracts from other Larrea species, such as *L. nitida* [18], the antifungal activity of *L. ameghinoi* is lower, likely due to differences in chemical composition. Although the observed values of antimicrobial activity are moderate, it is possible and expected that the ongoing partitioning process of the extracts and the subsequent isolation of the major compounds will allow the identification of molecules that show better values with respect to the extracts of origin.

EMLaMAQ and EMLaSAO show a significant content of malic acid (compound **2**), recognized for its important role in energy metabolism. Its potential capacity to intervene in the regulation of redox balance and cellular signaling links it to the prevention of cardiovascular diseases and conditions related to oxidative stress. Additionally, its antimicrobial properties support its potential as a versatile metabolite for the therapy of various pathologies [36]. Regarding carbohydrate-hydrolyzing enzymes, oleanane-type triterpenes provide a plausible explanation for the observed α-glucosidase and α-amylase inhibition: oleanolic acid (compound **20**) and its derivatives have been shown to significantly inhibit α-glucosidase in vitro, consistent with the IC_50_ values obtained in our assays [37]. The antimicrobial activity of oleanolic acid has also been reported [38]. In summary, the results support that, unlike other Larrea species whose chemical profile is mainly associated with a high concentration of lignans such as NDGA, its isomers and derivatives, and flavonoids as major constituents, *L. ameghinoi* presents a distinctive combination of predominant polyphenols and a low concentration of NDGA and some of its isomers. This particular composition, associated with antioxidant, anticholinesterase, and α-glucosidase inhibitory activities, as well as antibacterial action against Gram-positive bacteria, represents the most probable chemical basis for the observed bioactive profile. The contrast in predominant chemistry suggests that *L. ameghinoi* may offer a different functional spectrum than its congeners, reinforcing its potential as a source of phytochemicals of interest for human health and guiding future research toward marker compound validation and bioactivity-guided fractionation specific to this species.

## 5. Conclusions

This study provides the first chemical and biological characterization of extracts from Larrea ameghinoi, a vulnerable medicinal species that grows in Argentine Patagonia. The results support the need to design a strategy to protect this species, given its potential as a source of antimicrobial, antioxidant, antitumor, and enzyme-inhibiting compounds of interest for human health. The results also support the medicinal use of this species in traditional Argentine medicine. These findings differentiate *L. ameghinoi* from other species in the genus, in which NDGA lignans and derivatives predominate, and suggest that its unique phytochemical composition could underlie its multitarget bioactivity. The results shown by *L ameghinoi* suggest potential and justify further research aimed at identifying the active principles responsible for the demonstrated biological activities. Bioactivity-guided isolation studies of the species’ major compounds are currently underway to confirm their contribution to the observed biological activities.

## Figures and Tables

**Table 1:** UHPLC-PDA-Q-TOF identification of metabolites from EMLaSAO and EMLaMAQ (Rt = Retention time in minutes).

Peak	Tentative Identification	[M-H]^−^	Rt (min.)	Measured Mass (*m*/*z*)	Theoretical Mass (*m*/*z*)	Accuracy (ppm)	Metabolite Type	MS Ions (ppm)
1	Na formiate (internal standard)	C_4_H_2_O_4_	0.37	112.9829	112.9856	3.1	Standard	-
2	Malic Acid	C_4_H_5_O_5_	0.99	133.01425	133.01461	2.69	Organic acid	96.95251
3	(1*R*,2*R*,3*R*,4*S*)-1,2,4-trihydro-1-(3,4-dihydroxyphenyl)-2,3-dimethyl-3,4,6,7-naphthalenetetrol	C_18_H_19_O_6_	4.46	331.1287	331.1187	−10.23	https://doi.org/10.3390/molecules26206186	663.2598 (2M-H), 184.0585
4	4-(2-acetyl-4,5-dihydroxyphenyl)-4-(3,4-dihydroxyphenyl)butan-2-one	C_18_H_17_O_6_	4.75	329.1131	329.1031	−30.4	https://doi.org/10.3390/molecules26206186	655.3461
5	Ribesin G9	C_27_H_29_O_5_	5.1	433.2144	433.2020	−28.4	tetrahydrofuranephenolic	184.0588, 327.1693, 301.1526, 867.4339,
6	Diosmetin	C_16_H_12_O_6_	5.2	299.0658	299.0561	−32.3	flavone	184.0590
7	(1R,2R,3R,4S)-4-(3,4-dihydroxyphenyl)-2,3-dimethyl-1,2,3,4-tetrahydronaphthalene-1,2,6,7-tetraol	C_18_H_17_O_6_	4.75	329.1131	329.1031	−30.4	https://doi.org/10.3390/molecules26206186	655.3461
8	Nordihidroguaiaretic Acid (NDGA)	C_18_H_21_O_4_	5.2	301.1541	301.1445	−32.6	Nordihidroguaiaretic Acid (NDGA)	184.0591, 603.31337
9	Ribesin G9 isomer	C_27_H_29_O_5_	5.2	433.2162	433.2020	−32.6	tetrahydrofuranephenolic	184.0588, 867.4358 (2M-H),
10	Ribesin G9 isomer	C_27_H_29_O_5_	5.4	433.2156	433.2020	−31.7	tetrahydrofuranephenolic	184.0595, 867.4344 (2M-H),
11	Nordihidroguaiaretic Acid (NDGA) isomer	C_18_H_21_O_4_	5.7	301.1541	301.1445	−32.6	Nordihidroguaiaretic Acid (NDGA)	184.0591, 603.31337
12	Gilvocarcin	C_27_H_25_O_9_	5.8	493.1658	493.1504	11.6	tetrahydrofuranephenolic	329.1136, 179.0414 (2M-H),
13	Ribesin G9 isomer	C_27_H_29_O_5_	5.4	433.2146	433.2020	6.5	tetrahydrofuranephenolic	184.0591, 867.4344 (2M-H),
14	Unknown	C_20_H_37_O_9_	6.9	421.2601	421.2880	−37.06	tetrahydrofuranephenolic	199.1774
15	Yangambin	C_24_H_30_O_8_	6.6	445.1791	445.1657	−43.5	Lignan	299.1379, 329.1106
16	Capillartemisin A	C_19_H_23_O_4_	8.3	315.1698	315.1602	−30.4	hydroxycinnamic acid	178.0328,134.0425
17	Unknown	C_22_H_41_O_9_	8.4	449.2866	449.2756	−27.5	hydroxycinnamic acid	403.1739, 227.2063
18	hydroxy Anacardic acid	C_22_H_29_O_4_	9.4	357.2184	357.2196	−34.8	hydroxylbenzoic acid derivative	245.1632
19	Anacardic acid	C_22_H_29_O_3_	9.3	341.2225	341.2333	−30.3	hydroxylbenzoic acid derivative	245.1632
20	Linolenic acid	C_18_H_29_O_2_	10.2	277.2264	277.2173	−32.7	Fatty acid	269.06408
21	Oleanolic acid	C_30_H_47_O_3_	10.6	455.3660	455.3531	−28.5	Triterpene	375.2864
22	Myristyl glucoside	C_20_H_39_O_6_	10.8	375.2870	375.2752	−31.4	Fatty acid	329.1102, 241.2275
23	Linoleic acid	C_18_H_31_O_2_	10.9	279.2413	279.2330	−30.0	Fatty acid	116.9323
24	Makisterone A	C_28_H_45_O_7_	11.2	493.3260	493.3171	−30.0	Terpene	237.0853
25	dehydroeburicoic acid	C_31_H_47_O_3_	11.3	467.3350	467.3531	38.6	Triterpene	295.2381, 631.3467
26	Unknown	C_42_H_45_O_5_	11.8	639.4107	639.3996	−11.17	Triterpene	603.4205
27	Unknown	C_56_H_87_O_14_	12.6	981.5919	981.5591	−34.4	Terpene	935.5900
28	Cucurbitacin B linoleyl ester	C_29_H_55_O_8_	13.2	531.3962	531.3902	−11.2	Terpene	463.3305
29	Cucurbitacin B linoleyl ester isomer	C_29_H_55_O_8_	13.5	531.3962	531.3902	−11.2	Terpene	463.3305
30	Unknown	C_56_H_87_O_14_	13.7	983.6155	983.6101	−5.5	Terpene	937.6060, 659.3766
31	Unknown	C_40_H_62_O_9_	13.9	685.4401	685.4262	−20.2	Terpene	345.2163
32	Unknown	C_50_H_75_O_9_	14.3	819.5574	819.5417	−2.8	Terpene	693.4756
33	Unknown	C_51_H_91_O_16_	14.9	959.6149	959.6173	178	Terpene	913.6076, 635.3786

**Table 2:** Antioxidant properties; total phenolics and flavonoids content of *L ameghinoi* extracts.

Assay	EMLaSAO	EMLaMAQ
Content of phenols		
Total phenolics (mg GAE/g extract)	239.50 ± 2.33	215.60 ± 1.98
Flavonoids (mg QE/g extract)	28.44 ± 1.29 ^a^	15.28 ± 0.29 ^b^
Antioxidant		
DPPH (EC_50_ in µg extract/mL)	10.10 ± 0.02	10.05 ± 0.01
FRAP (mgETrolox/ g extract;)	28.94 ± 2.04	28.70 ± 1.80
TEAC (mgETrolox/g extract;)	54.84 ± 0.03	54.88 ± 0.05

No significant differences were found between the samples in the different trials, except for flavonoids using an analysis of variance (ANOVA) followed by Dunnett’s comparison test (significance *p* < 0.05). Different letters in flavonoids indicate statistically significant differences (*p* < 0.05) between the samples.

**Table 3:** Cholinesterases, Amylase, and Glucosidase Enzyme Inhibitory Activities of EMLaSAO and EMLaMAQ.

Sample	AChE ^1^	BChE ^1^	Amylase ^1^	Glucosidas e ^1^
EMLaSAO	108.0 ± 0.9 ^a^	50.4 ± 0.4	478.0 ± 0.2 ^a^	63.8 ± 0.2 ^a^
EMLaMAQ	188.0 ± 0.6 ^b^	49.6 ± 0.4	589.4 ± 0.9 ^b^	122.3 ± 0.1 ^b^
Galantamine	0.45 ± 0.02			
Acarbose	1.33 ± 0.02		10.05 ± 0.02	138.8 ± 0.02

^1^ IC_50_ values are expressed in µg/mL and were calculated as means ± SD. Different letters show statistically significant differences (*p* < 0.05) between the samples in the different trials, using ANOVA (analysis of variance) followed by Dunnett’s comparison test.

**Table 4:** Antimicrobial activity of *L. ameghinoi* and antibiotic references (MICs and MBCs in µg extract/mL).

Bacteria/Yeast	Extract				Antibiotic References			
	EMLaSAO		EMLaMAQ		Cefotaxime		Imipecil	
Gram (+)	MIC	MBC	MIC	MBC	MIC	MBC	MIC	MBC
*MSSA Staphylococcus aureus* methicillin-sensitive ATCC 25923	125	250	>1000	>1000	0.5	0.5	0.5	0.5
*MRSA S. aureus* methicillin-resistant ATCC 43300	125	>1000	>1000	>1000	0.5	0.5	0.5	0.5
*Staphylococcus aureus-* MQ1	125	>1000	>1000	>1000	0.8	1	1	1
*Staphylococcus aureus-* MQ2	125	250	>1000	>1000	0.25	0.5	0.5	0.5
Gram (−)								
*Escherichia coli* ATCC 25922	1000	>1000	>1000	>1000	1.9	1.9	0.5	1
*Salmonella sp.*	1000	>1000	>1000	>1000	1	1	0.5	1
Yeast	MIC	MFC	MIC	MFC			Ketoconazole	
*Candida albicans-MQ1924*	500	>1000	1000	>1000			1	1
*C. glabrata-MQ1*	>1000	>1000	1000	>1000			1	1.5
*C. tropicalis-C131*	125	500	250	>1000			1.5	1.5
*C. tropicalis-MQ1*	500	>1000	>1000	>1000			0.625	0.625
*C. parapsilopsis-MQ1*	250	>1000	500	>1000			0.156	0.156
*Cryptococcus neoformans* *ATCC 32264*	>1000	>1000	>1000	>1000			0.625	2.5

MIC: Minimum inhibitory concentration, MBC: Minimum Bactericidal Concentration.

## Data Availability

Data are contained within the article. The assay protocols are available in detail upon request from the authors.
